# Supplementary material for: High-Fidelity 3D Stray Magnetic Field Mapping of Smartphones to Address Safety Considerations with Active Implantable Electronic Medical Devices
Source: Sensors (Basel). 2023 Jan 20;23(3):1209. doi: 10.3390/s23031209 (PMC9919430; doi:10.3390/s23031209)
Supplement: Supplementary file 1 [file sensors-23-01209-s001.zip › sensors-2134360-supplementary.pdf]

Supplementary Materials for

High-Fidelity 3D Stray Magnetic Field Mapping of Smartphones to Address Safety Considerations with Active Implantable Electronic Medical Devices

Nandita Saha [1,2], Jason M. Millward [1], Carl J. J. Herrmann [1,3], Faezeh Rahimi [1,4], Haopeng Han [1], Philipp Lacour [5], Florian Blaschke [5] and Thoralf Niendorf [1,2,*]

[1] Max-Delbrück-Center for Molecular Medicine in the Helmholtz Association (MDC), Berlin Ultrahigh Field Facility (B.U.F.F.), 13125 Berlin, Germany
[2] Experimental and Clinical Research Center (ECRC), A Joint Cooperation between the Charité Medical Faculty and the Max-Delbrück Center for Molecular Medicine in the Helmholtz Association, 13125 Berlin, Germany
[3] Department of Physics, Humboldt University of Berlin, 10117 Berlin, Germany
[4] Chair of Medical Engineering, Technische Universität Berlin, 10623 Berlin, Germany
[5] Department of Cardiology, Charité—Universitätsmedizin Berlin, Campus Virchow-Klinikum, 13353 Berlin, Germany
* Correspondence: thoralf.niendorf@mdc-berlin.de

This PDF file includes:
Supplementary Movie Legends

Supplementary Materials for this manuscript include the following: Movies S1 to S4

Supplementary Movie Legends

Movie S1: Setup used for mapping the static stray magnetic field of the iPhone 13 Pro, iPhone 12, and the MagSafe wireless charger. For spatial mapping of the stray magnetic field, COSI Measure was used to control the sample trajectory of the Hall probe. The Hall probe was placed perpendicular to the surface plane of the iPhone 13 Pro and iPhone 12.

Movie S2: The 3D distribution of stray magnetic fields obtained from the circular array of magnets (back, front), cameras (back, front), and speakers (top, bottom) of the iPhone 13 Pro.

Movie S3: The 3D distribution of stray magnetic fields obtained from the circular array of magnets (back, front), cameras (back, front), and speakers (top, bottom) of the iPhone 12.

Movie S4: The 3D distribution of stray magnetic fields obtained from the MagSafe charger, MagSafe + iPhone 13 Pro, and MagSafe + iPhone 12.
